# Supplementary material for: Family-Centered Design: Interactive Performance Testing and User Interface Evaluation of the Slovenian eDavki Public Tax Portal
Source: Sensors (Basel). 2021 Jul 30;21(15):5161. doi: 10.3390/s21155161 (PMC8348796; doi:10.3390/s21155161)
Supplement: Supplementary file 1 [file sensors-21-05161-s001.zip › sensors-1282635-supplementary.pdf]

sensors MDPI

Supplementary Materials

Family-Centered Design: Interactive Performance Testing and User Interface Evaluation of the Slovenian eDavki Public Tax Portal

Jure Trilar *, Tjaša Sobočan and Emilija Stojmenova Duh

ICT Department, Faculty of Electrical Engineering, University of Ljubljana, 1000 Ljubljana, Slovenia; Tjasa.Sobocan@ltfe.org (T.S.); emilija.stojmenova@fe.uni-lj.si (E.S.D.)
* Correspondence: jure.trilar@fe.uni-lj.si

1. Testing platform

Table S1. Testing timeframe and responses.

First start	2021-04-12 12:19:15
Last finish	2021-04-26 18:01:36
Duration	14 days
Participants	152

Table S2. Measured cumulatives.

	Interface 1	Interface 2
Total time	14,952,966 ms	12,576,724 ms
Clicks	1640	1598
First starts (order)	74	78

Table S3. Questionnaires collected.

	Interface 1	Interface 2
SUS	73	75
UEQ	70	75
NASA-TLX	70	74

Table S4. Operating System.

OS	N	%
Windows	113	74.34%
Mac OS	26	17.11%
Linux	13	8.55%

Table S5. Screen resolution => Frequency.

1920x1080 => 41	1280x1024 => 4	2752x1152 => 1	820x1180 =>1
1920x1200 => 26	1280x720 => 4	3072x1728 => 1	2294x960 =>1
1536x864 => 15	2048x1152 => 3	1792x1120 => 1	1979x1237 =>1
1366x768 => 10	2560x1080 => 2	412x857 => 1	1536x960 =>1
1680x1050 =>8	3360x1890 =>2	1876x1173 => 1	1093x615 => 1
1600x900 =>7	1280x800 =>2	1529x640 => 1	1024x819 => 1
1440x900 =>6	2560x1440 => 1	2872x1203 => 1	412x892 => 1
3440x1440 => 5	1344x840 => 1	768x1024 => 1	

Calculated average screen, viewport size: 1809x1048 pixels.

Table S6. Testing satisfaction core. Scale: 1-not satisfied, 5-very satisfied.

	Mean	N	Std. Dev
Satisfaction [1..5]	4.4615	52	0.8561

Table S7. Familiarity with Slovenian eDavki Tax portal. Scale: 1-none, 2-somewhat familiar, 3-using sporadically, 4-using regularly.

	Mean	N	Std. Dev
Familiarity [1..4]	2.8214	112	0.8785

2. Demography

Table S8. Gender.

	N	%
Male	71	46.7105%
Female	40	26.3158%
Unknown	41	26.9737%

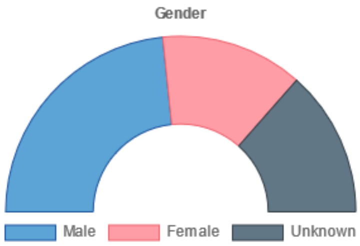

Figure S1. Gender distribution graph.

Table S9. Age and generation.

	N	%	Min	Max
Age (all w/o missing)	114	100%	23	85
Gen 1 (18–64)	86	75.438596491228%	23	62
Gen 2 (65+)	28	24.561403508772%	65	85

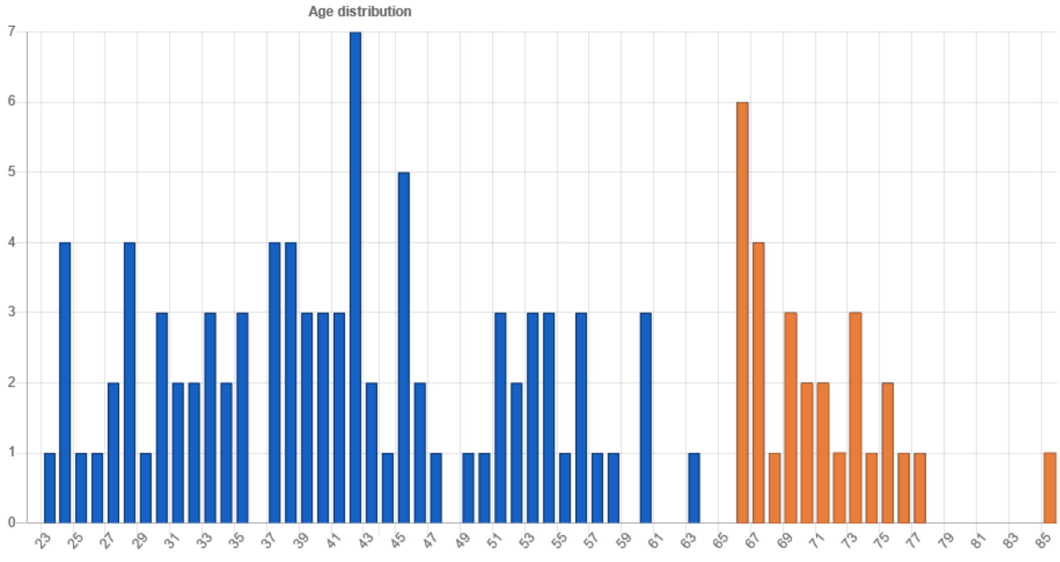

Figure S2. Age distribution chart.

Table S10. Developmental goals question set vs. age based generation classification.

	Fit (where age based generation = dev. goals based generation) %
All	80.53%
Gen 1 = Age 18–64	83.53%
Gen 2 = Age 65+	71.43%

3. Normality tests on samples

Table S11. Shapiro-Wilk formal test for normal distribution on samples within Interface 1 and Interface 2.

		Interface 1 samples			Interface 2 samples		
		Shapiro Wilk test for normal distribution of samples					
		W	p-value	Result (α = 0.05)	W	p-value	Result (α = 0.05)
Performance metrics							
**Success rate**		0.459998	0.000000	**0**	0.425303	0.000000	**0**
**Time on task (ms)**		0.621031	6.66134×10^{-16}	**0**	0.657893	3.33067×10^{-16}	**0**
User evaluation							
	**SUS**	0.956919	0.0138985	**0**	0.959773	0.0167003	**0**
**UEQ**	Attractiveness	0.961109	0.0288360	**0**	0.960028	0.0183942	**0**
	Perspicuity	0.952945	0.0103726	**0**	0.949040	0.00441825	**0**
	Efficiency	0.980272	0.336408	**1**	0.966096	0.0419344	**0**
	Dependability	0.968541	0.0753574	**1**	0.965148	0.0368175	**0**
	Stimulation	0.956084	0.0152913	**0**	0.955466	0.0100609	**0**
	Novelty	0.955833	0.0148215	**0**	0.969566	0.0677256	**1**
**NASA-TLX**	Mental	0.950850	0.950850	**0**	0.952363	0.00723613	**0**
	Physical	0.862331	0.00000168307	**0**	0.864696	0.00000116408	**0**
	Temporal	0.960003	0.0250446	**0**	0.935711	0.000969826	**0**
	Performance	0.893850	0.0000223519	**0**	0.878830	0.00000369816	**0**
	Effort	0.943684	0.00343298	**0**	0.939527	0.00150944	**0**
	Frustration	0.944909	0.00395993	**0**	0.937016	0.00112694	**0**

Table S12. Shapiro-Wilk formal test for normal distribution on samples within Generation 1 and Generation 2.

		Generation 1 (18-64 years) samples			Generation 2 (65+ years) samples		
		Shapiro Wilk test for normal distribution of samples					
		W	p-value	Result (α=0.05)	W	p-value	Result (α=0.05)
Performance metrics							
**Success rate**		0.704504	2.22045×10^{-16}	**0**	0.752580	2.29789×10^{-10}	**0**
**Time on task (ms)**		0.519431	0.00000	**0**	0.701698	1.54325×10^{-11}	**0**
User evaluation							
	**SUS**	0.968550	0.136717	**1**	0.792181	0.000130029	**0**
**UEQ**	Attractiveness	0.943371	0.00995052	**0**	0.925620	0.0540142	**1**
	Perspicuity	0.917141	0.000827075	**0**	0.887896	0.00848780	**0**
	Efficiency	0.959074	0.0515198	**1**	0.897900	0.0140577	**0**
	Dependability	0.916087	0.000753462	**0**	0.814195	0.000310031	**0**
	Stimulation	0.896596	0.000146051	**0**	0.884160	0.00705592	**0**
	Novelty	0.883654	0.0000531739	**0**	0.926051	0.0624379	**1**
**NASA-TLX**	Mental	0.970761	0.190398	**1**	0.971064	0.630139	**1**
	Physical	0.885811	0.0000720023	**0**	0.905511	0.0208291	**0**
	Temporal	0.980986	0.518912	**1**	0.962829	0.450261	**1**
	Performance	0.925173	0.00190330	**0**	0.888781	0.00887014	**0**
	Effort	0.977057	0.360964	**1**	0.973571	0.717010	**1**
	Frustration	0.971814	0.212517	**1**	0.897680	0.0139004	**0**

4. Interfaces global

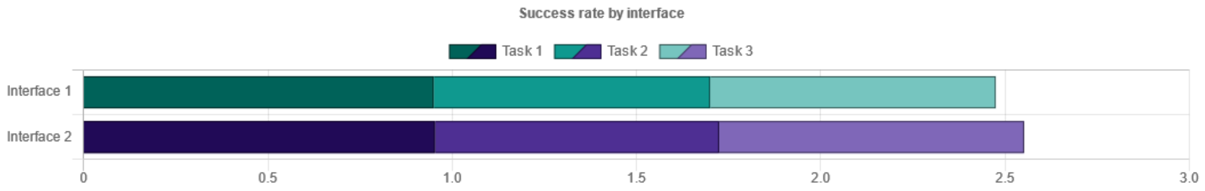

Figure S3. Task success rate chart stacked by interface.

Table S13. Success rate by interface.

	Interface 1						Interface 2						Mann-Whitney U test	
	Mean	Std. Dev.	N	Conf.	Conf. min.	Conf. max.	Mean	Std. Dev.	N	Conf.	Conf. min.	Conf. max.	P-value	H_0 rejection (α=0.05)
Task 1	0.95	0.2193	80	0.0481	0.9019	0.9981	0.954	0.2106	87	0.0443	0.9098	0.9983	0.9069	0
Task 2	0.75	0.4357	80	0.0955	0.6545	0.8455	0.7701	0.4232	87	0.0889	0.6812	0.859	0.7633	0
Task 3	0.775	0.4202	80	0.0921	0.6829	0.8671	0.8276	0.3799	87	0.0798	0.7478	0.9074	0.3966	0
All	0.825	0.3808	240	0.0482	0.7768	0.8732	0.8506	0.3572	261	0.0433	0.8072	0.8939	0.4380	0

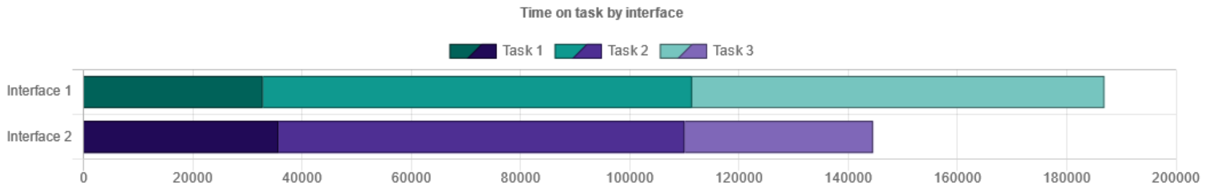

Figure S4. Time on task rate chart stacked by interface.

Table S14. Time on task by interface.

	Interface 1						Interface 2						Mann-Whitney U test	
	Mean	Std. Dev.	N	Conf.	Conf. min.	Conf. max.	Mean	Std. Dev.	N	Conf.	Conf. min.	Conf. max.	P-value	H_0 rejection (α=0.05)
Task 1	32,829.1	49,707.4971	80	10,892.6356	21,936.4644	43,721.7356	35,655.7586	61,211.6964	87	12,862.6621	22,793.0965	48,518.4207	0.5805	0
Task 2	78,564.8375	98,171.977	80	21,512.883	57,051.9545	100,077.7205	74,403.0345	76,652.2793	87	16,107.2544	58,295.7801	90,510.2889	0.8993	0
Task 3	75,518.1375	157,843.566	80	34,588.9964	40,929.1411	110,107.1339	34,501.2529	27,902.5466	87	5,863.2753	28,637.9776	40,364.5282	0.0005553	1
All	62,304.025	112,585.6953	240	14,244.0557	48,059.9693	76,548.0807	48,186.682	61,526.6014	261	7,464.466	40,722.216	55,651.148	0.02112	1

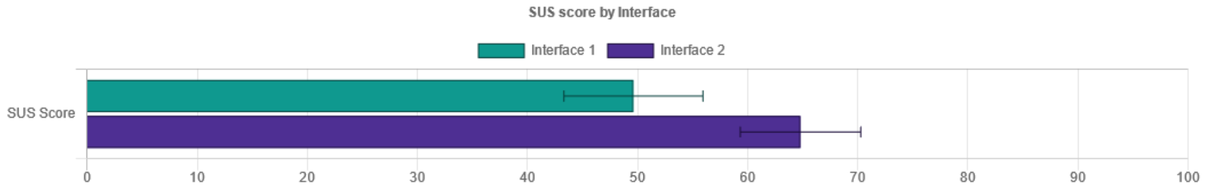

Figure S5. SUS score by interface chart.

Table S15. SUS score by interface.

	Interface 1						Interface 2						Mann-Whitney U test	
	Mean	Std. Dev.	N	Conf.	Conf. min.	Conf. max.	Mean	Std. Dev.	N	Conf.	Conf. min.	Conf. max.	P-value	H_0 rejection (α=0.05)
SUS Score	49.6233	27.5242	73	6.3141	43.3092	55.9374	64.8	24.1678	75	5.4697	59.3303	70.2697	0.001044	1

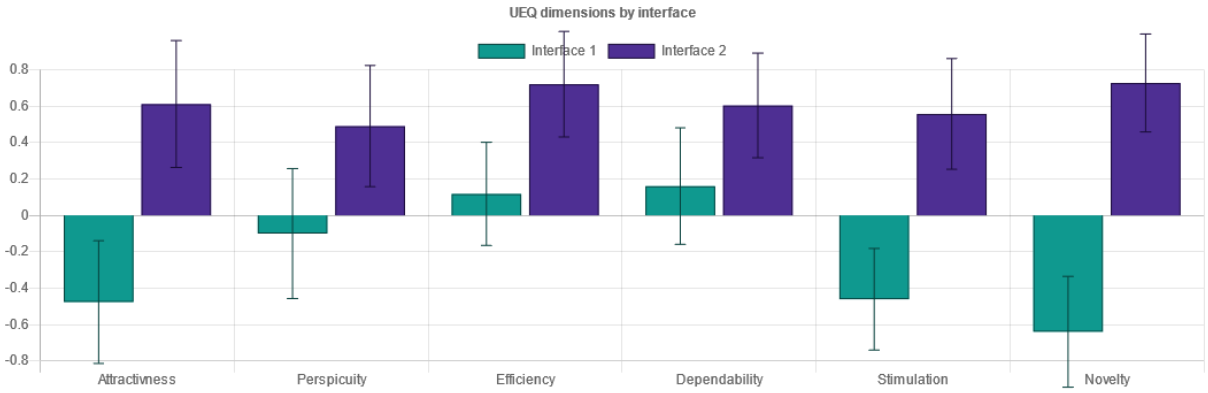

Figure S6. UEQ results by interface chart.

Table S16. UEQ results by interface.

Item	Interface 1						Interface 2						Mann-Whitney U test	
	Mean	Std. Dev.	N	Conf.	Conf. min.	Conf. max.	Mean	Std. Dev.	N	Conf.	Conf. min.	Conf. max.	P-value	H_0 rejection (α=0.05)
Attractivness	−0.4762	1.4386	70	0.337	−0.8132	−0.1392	0.6111	1.5396	75	0.3484	0.2627	0.9596	0.00004424	1
Perspicuity	−0.1	1.523	70	0.3568	−0.4568	0.2568	0.49	1.469	75	0.3325	0.1575	0.8225	0.05805	0
Efficiency	0.1179	1.2082	70	0.283	−0.1652	0.4009	0.72	1.2807	75	0.2898	0.4302	1.0098	0.002178	1
Dependability	0.1607	1.3647	70	0.3197	−0.159	0.4804	0.6033	1.2688	75	0.2872	0.3162	0.8905	0.07301	0
Stimulation	−0.4607	1.19	70	0.2788	−0.7395	−0.1819	0.5567	1.3423	75	0.3038	0.2529	0.8604	0.00004006	1
Novelty	−0.6393	1.2991	70	0.3043	−0.9436	−0.3349	0.7267	1.1878	75	0.2688	0.4579	0.9955	8.112×10^{-9}	1

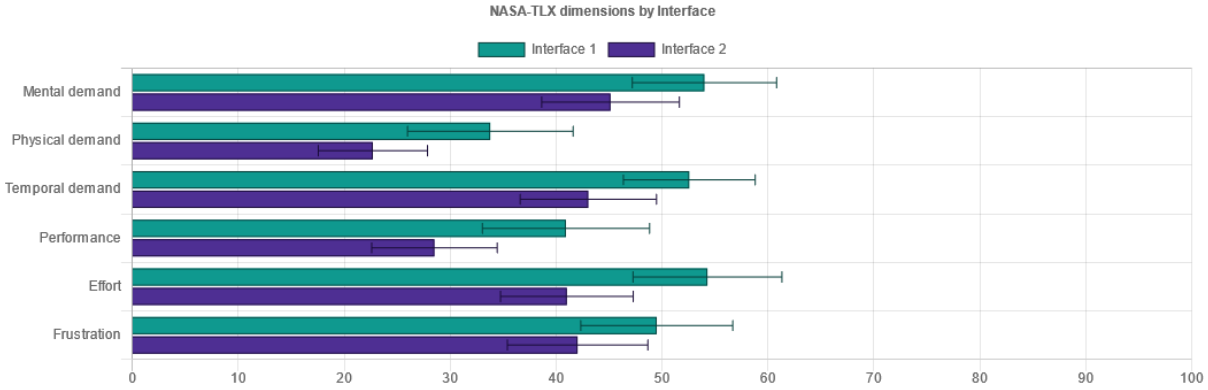

Figure S7. NASA-Task Load Index results by interface chart.

Table S17. NASA-Task Load results by interface.

Item	Interface 1						Interface 2						Mann-Whitney U test	
	Mean	Std. Dev.	N	Conf.	Conf. min.	Conf. max.	Mean	Std. Dev.	N	Conf.	Conf. min.	Conf. max.	P-value	H_0 rejection (α=0.05)
Mental demand	54	29.0751	70	6.8113	47.1887	60.8113	45.1351	28.5251	74	6.4993	38.6358	51.6344	0.05798	0
Physical demand	33.7857	33.367	70	7.8167	25.969	41.6024	22.7027	22.6224	74	5.1544	17.5483	27.8571	0.1228	0
Temporal demand	52.5714	26.5633	70	6.2229	46.3486	58.7943	43.0405	28.2457	74	6.4357	36.6049	49.4762	0.05035	0
Performance	40.9286	33.7004	70	7.8948	33.0337	48.8234	28.5135	25.997	74	5.9233	22.5902	34.4368	0.05756	1
Effort	54.2857	30.0035	70	7.0288	47.257	61.3145	41.0135	27.4982	74	6.2653	34.7482	47.2788	0.00692	1
Frustration	49.5	30.6234	70	7.174	42.326	56.674	42.027	29.1068	74	6.6318	35.3952	48.6589	0.1599	0

5. Generations global

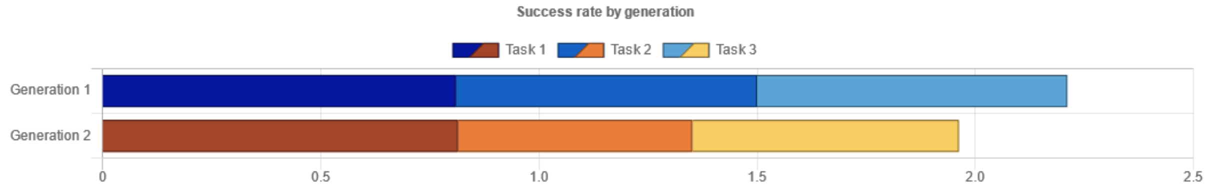

Figure S8. Task success rate chart stacked by generation.

Table S18. Success rate by generation.

	Generation 1						Generation 2						Mann-Whitney U test	
	Mean	Std. Dev.	N	Conf.	Conf. min.	Conf. max.	Mean	Std. Dev.	N	Conf.	Conf. min.	Conf. max.	P-value	H_0 rejection (α=0.05)
Task 1	0.8099	0.2849	71	0.0663	0.7436	0.8761	0.8148	0.3439	27	0.1297	0.6851	0.9445	0.6290	0
Task 2	0.6901	0.3814	71	0.0887	0.6014	0.7789	0.537	0.4143	27	0.1563	0.3808	0.6933	0.04754	1
Task 3	0.7113	0.3748	71	0.0872	0.6241	0.7984	0.6111	0.3755	27	0.1417	0.4695	0.7528	0.1886	0
All	0.7371	0.3521	213	0.0473	0.6898	0.7844	0.6543	0.3924	81	0.0855	0.5689	0.7398	0.01021	1

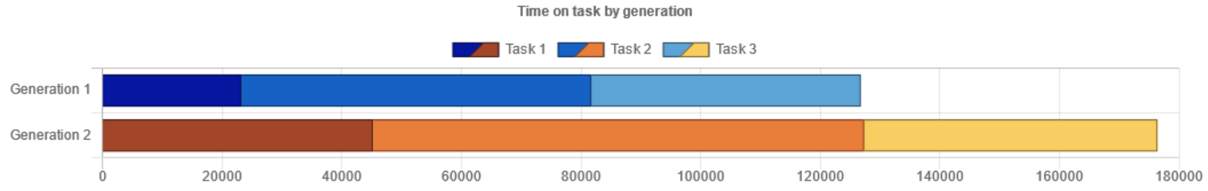

Figure S9. Time on task rate chart stacked by generation.

Table S19. Time on task by generation.

	Generation 1						Generation 2						Mann-Whitney U test	
	Mean	Std. Dev.	N	Conf.	Conf. min.	Conf. max.	Mean	Std. Dev.	N	Conf.	Conf. min.	Conf. max.	P-value	H_0 rejection (α=0.05)
Task 1	23,153.7746	32,132.148	71	7,474.2334	15,679.5413	30,628.008	45,195.2407	58,601.3752	27	22,104.5663	23,090.6745	67,299.807	0.00001916	1
Task 2	58,604.4366	52,027.1281	71	12,101.9889	46,502.4477	70,706.4255	82,155.9444	85,350.5462	27	32,194.4117	49,961.5327	11,4350.3562	0.4571	0
Task 3	45,023.5	83,842.1178	71	19,502.4484	25,521.0516	64,525.9484	49,068.9444	37,964.7628	27	14,320.3911	34,748.5533	63,389.3356	0.2406	0
All	42,260.5704	61,400.4579	213	8,245.8965	34,014.6739	50,506.4669	58,806.7099	65,042.9476	81	14,164.9086	44,641.8013	72,971.6185	0.003251	1

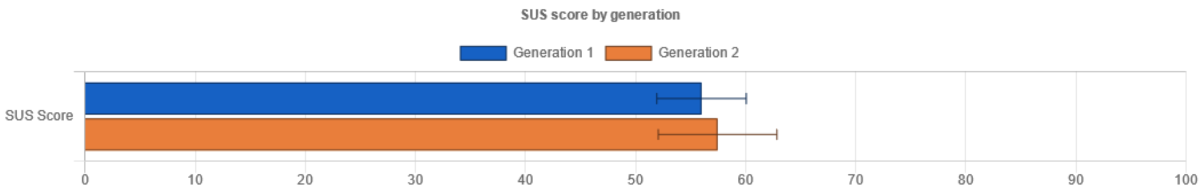

Figure S10. SUS score by generation chart.

Table S20. SUS score by generation.

	Generation 1						Generation 2						Mann-Whitney U test	
	Mean	Std. Dev.	N	Conf.	Conf. min.	Conf. max.	Mean	Std. Dev.	N	Conf.	Conf. min.	Conf. max.	P-value	H_0 rejection (α=0.05)
SUS Score	55.9914	15.7832	58	4.062	51.9294	60.0534	57.4519	14.02	26	5.3891	52.0628	62.841	0.7861	0

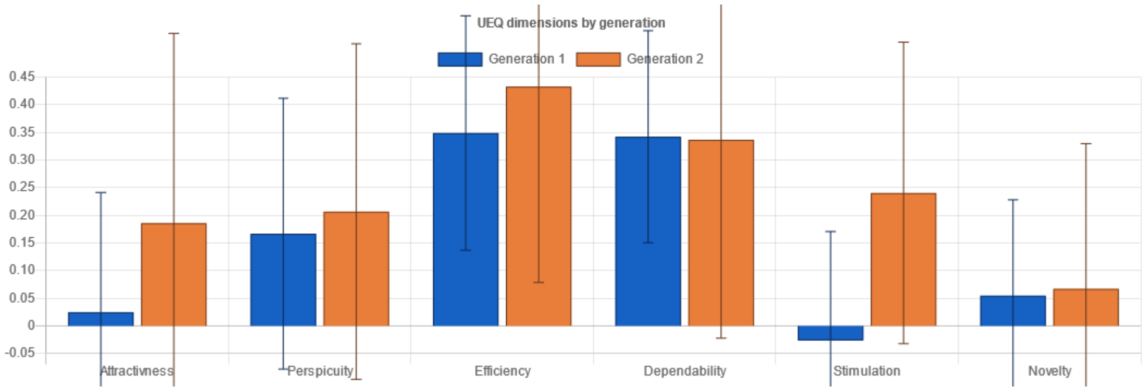

Figure S11. UEQ results by generation chart.

Table S21. UEQ results by generation.

Item	Generation 1						Generation 2						Mann-Whitney U Test	
	**Mean**	**Std. Dev.**	**N**	**Conf.**	**Conf. min.**	**Conf. max.**	**Mean**	**Std. Dev.**	**N**	**Conf.**	**Conf. min.**	**Conf. max.**	**P-value**	**H_0 rejection (α=0.05)**
Attractivness	0.0249	0.833	57	0.2163	−0.1914	0.2411	0.1859	0.892	26	0.3429	−0.157	0.5288	0.5446	0
Perspicuity	0.1667	0.9429	57	0.2448	−0.0781	0.4115	0.2067	0.7889	26	0.3033	−0.0965	0.51	0.9331	0
Efficiency	0.3487	0.8162	57	0.2119	0.1368	0.5606	0.4327	0.9214	26	0.3542	0.0785	0.7869	0.5707	0
Dependability	0.3421	0.7382	57	0.1916	0.1505	0.5337	0.3365	0.9333	26	0.3587	−0.0222	0.6953	0.7929	0
Stimulation	−0.0263	0.7584	57	0.1969	−0.2232	0.1706	0.2404	0.7088	26	0.2725	−0.0321	0.5128	0.02848	1
Novelty	0.0548	0.6673	57	0.1732	−0.1184	0.2281	0.0673	0.6821	26	0.2622	−0.1949	0.3295	0.57	0

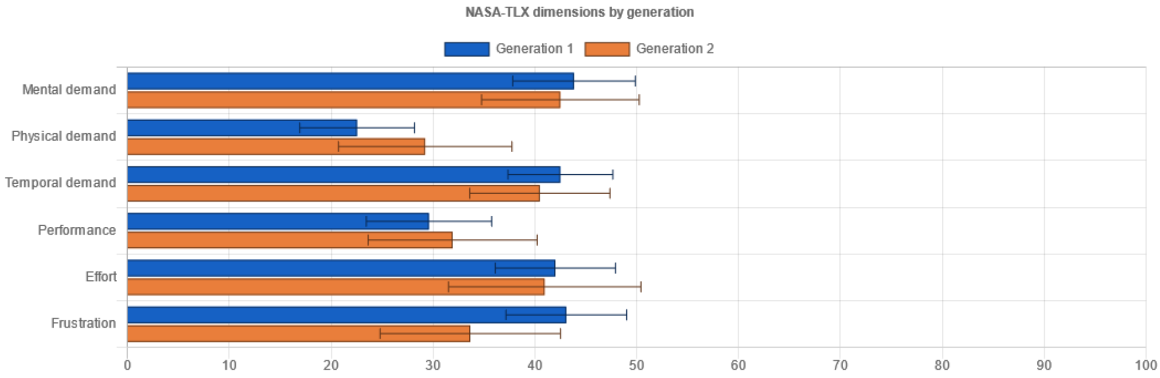

Figure S12. NASA-Task Load Index results by generation chart.

Table S22. NASA-Task Load results by generation.

Item	Generation 1						Generation 2						Mann-Whitney U test	
	Mean	Std. Dev.	N	Conf.	Conf. min.	Conf. max.	Mean	Std. Dev.	N	Conf.	Conf. min.	Conf. max.	P-value	H_0 rejection (α=0.05)
Mental demand	43.8393	22.9771	56	6.0181	37.8212	49.8574	42.5	20.137	26	7.7404	34.7596	50.2404	0.9801	0
Physical demand	22.5446	21.5031	56	5.632	16.9126	28.1767	29.2308	22.1559	26	8.5165	20.7143	37.7472	0.1205	0
Temporal demand	42.5	19.6561	56	5.1483	37.3517	47.6483	40.4808	17.9028	26	6.8816	33.5992	47.3624	0.7602	0
Performance	29.5982	23.4946	56	6.1536	23.4446	35.7518	31.9231	21.579	26	8.2947	23.6284	40.2178	0.6852	0
Effort	42.0089	22.5349	56	5.9023	36.1067	47.9112	40.9615	24.5772	26	9.4472	31.5144	50.4087	0.8692	0
Frustration	43.0804	22.6284	56	5.9267	37.1536	49.0071	33.6538	23.0459	26	8.8586	24.7953	42.5124	0.03818	1

6. Generation 1 (0-64 years old) by Interfaces

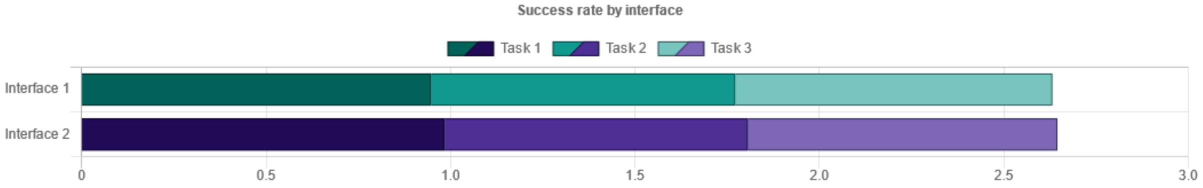

Figure S13. Task success rate chart stacked by interface for Generation 1.

Table S23. Success rate by interface for Generation 1.

	Interface 1						Interface 2						Mann-Whitney U test	
	Mean	Std. Dev.	N	Conf.	Conf. min.	Conf. max.	Mean	Std. Dev.	N	Conf.	Conf. min.	Conf. max.	P-value	H_0 rejection (α=0.05)
Task 1	0.9474	0.2253	57	0.0585	0.8889	1.0059	0.9839	0.127	62	0.0316	0.9523	1.0155	0.2755	0
Task 2	0.8246	0.3837	57	0.0996	0.7249	0.9242	0.8226	0.3851	62	0.0959	0.7267	0.9185	0.9807	0
Task 3	0.8596	0.3504	57	0.091	0.7687	0.9506	0.8387	0.3708	62	0.0923	0.7464	0.931	0.7544	0
All	0.8772	0.3292	171	0.0493	0.8279	0.9265	0.8817	0.3238	186	0.0465	0.8352	0.9283	0.8964	0

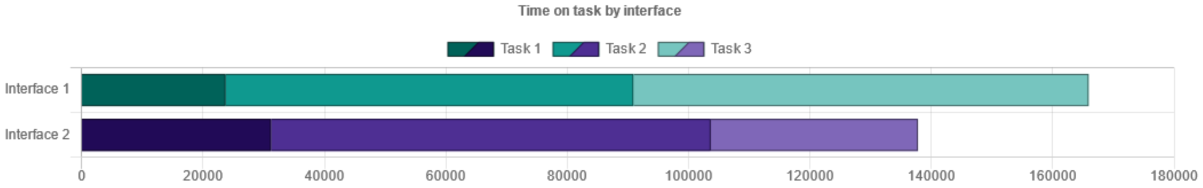

Figure S14. Time on task rate chart stacked by interface for Generation 1.

Table S24. Time on task by interface for Generation 1.

	Interface 1						Interface 2						Mann-Whitney U test	
	Mean	Std. Dev.	N	Conf.	Conf. min.	Conf. max.	Mean	Std. Dev.	N	Conf.	Conf. min.	Conf. max.	P-value	H_0 rejection (α=0.05)
Task 1	23,717.6842	23,045.7753	57	5,982.8755	17,734.8087	29,700.5597	31,224.6452	65,222.9093	62	16,235.3028	14,989.3423	47,459.948	0.7678	0
Task 2	67,215.5088	85,927.6448	57	22,307.5334	44,907.9754	89,523.0422	72,428.1613	60,204.4881	62	14,986.1162	57,442.0451	87,414.2775	1.938×10^{-7}	1
Task 3	75,014.5965	182,396.3249	57	47,351.6075	27,662.989	122,366.204	34,153.3065	26,791.5876	62	6,668.9687	27,484.3378	40,822.2751	0.006499	1
All	55,315.9298	118,653.0736	171	17,784.3095	37,531.6203	73,100.2394	45,935.371	56,469.0708	186	8,115.4058	37,819.9652	54,050.7768	0.4337	0

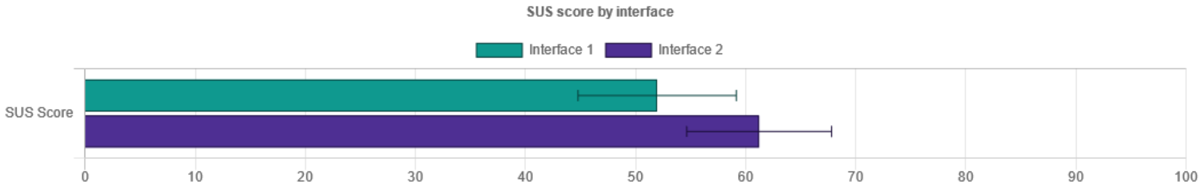

Figure S15. SUS score by interface chart for Generation 1.

Table S25. SUS score by interface for Generation 1.

	Interface 1						Interface 2						Mann-Whitney U test	
	Mean	Std. Dev.	N	Conf.	Conf. min.	Conf. max.	Mean	Std. Dev.	N	Conf.	Conf. min.	Conf. max.	P-value	H_0 rejection (α=0.05)
SUS Score	51.9608	26.1931	51	7.1888	44.772	59.1496	61.2264	24.4341	53	6.5783	54.6481	67.8047	0.0834	0

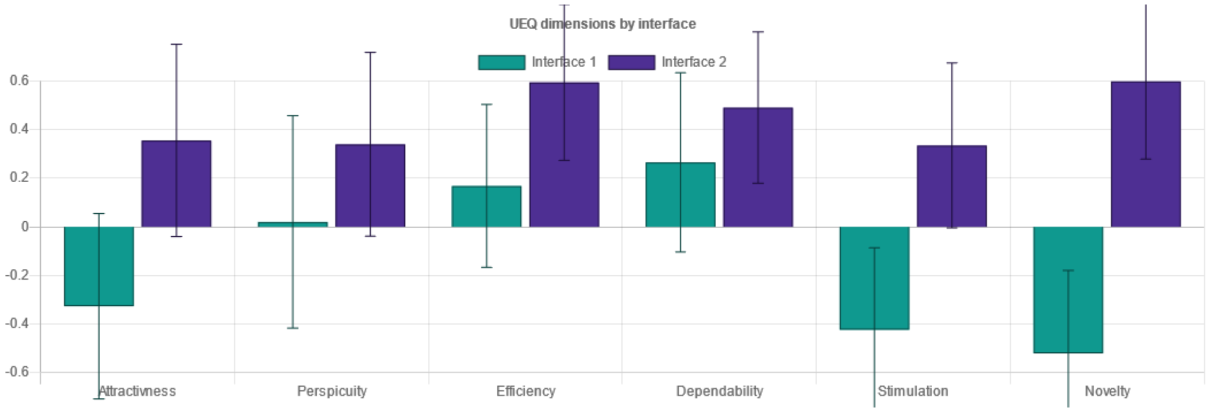

Figure S16. UEQ results by interface chart for Generation 1.

Table S26. UEQ results by interface for Generation 1.

Item	Interface 1						Interface 2						Mann-Whitney U test	
	Mean	Std. Dev.	N	Conf.	Conf. min.	Conf. max.	Mean	Std. Dev.	N	Conf.	Conf. min.	Conf. max.	P-value	H_0 rejection (α=0.05)
Attractivness	−0.3265	1.3612	49	0.3811	−0.7077	0.0546	0.3553	1.4687	53	0.3954	−0.0401	0.7508	0.01971	1
Perspicuity	0.0204	1.5603	49	0.4369	−0.4165	0.4573	0.3396	1.4032	53	0.3778	−0.0382	0.7174	0.5165	0
Efficiency	0.1684	1.1961	49	0.3349	−0.1665	0.5033	0.5943	1.1911	53	0.3207	0.2737	0.915	0.06721	0
Dependability	0.2653	1.3164	49	0.3686	−0.1033	0.6339	0.4906	1.156	53	0.3112	0.1793	0.8018	0.3961	0
Stimulation	−0.4235	1.2057	49	0.3376	−0.7611	−0.0859	0.3349	1.2595	53	0.3391	−0.0042	0.674	0.007306	1
Novelty	−0.5204	1.2182	49	0.3411	−0.8615	−0.1793	0.5991	1.1892	53	0.3202	0.2789	0.9192	0.00002204	1

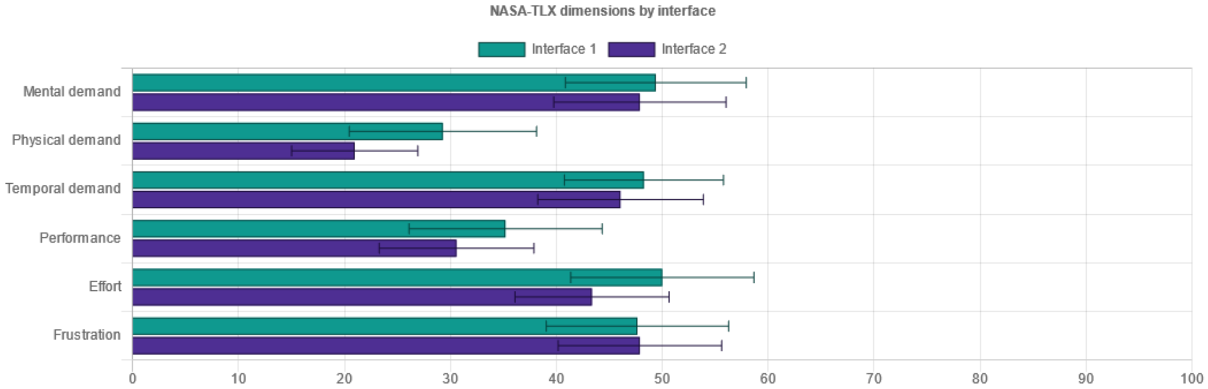

Figure S17. NASA-Task Load Index results by interface chart for Generation 1.

Table S27. NASA-Task Load results by interface for Generation 1.

Item	Interface 1						Interface 2						Mann-Whitney U test	
	Mean	Std. Dev.	N	Conf.	Conf. min.	Conf. max.	Mean	Std. Dev.	N	Conf.	Conf. min.	Conf. max.	P-value	H_0 rejection (α=0.05)
Mental demand	49.3878	30.493	49	8.5381	40.8497	57.9258	47.8846	29.9239	52	8.1334	39.7512	56.018	0.7849	0
Physical demand	29.2857	31.5733	49	8.8405	20.4452	38.1262	20.9615	21.9187	52	5.9576	15.004	26.9191	0.3923	0
Temporal demand	48.2653	26.8202	49	7.5097	40.7556	55.775	46.0577	28.7541	52	7.8155	38.2422	53.8732	0.7407	0
Performance	35.2041	32.5634	49	9.1177	26.0863	44.3218	30.5769	26.874	52	7.3044	23.2725	37.8813	0.7712	0
Effort	50	30.8896	49	8.6491	41.3509	58.6491	43.3654	26.7655	52	7.275	36.0904	50.6403	0.2985	0
Frustration	47.6531	30.7392	49	8.607	39.0461	56.26	47.8846	28.394	52	7.7176	40.167	55.6022	0.8942	0

7. Generation 2 (65+ years old) by Interfaces

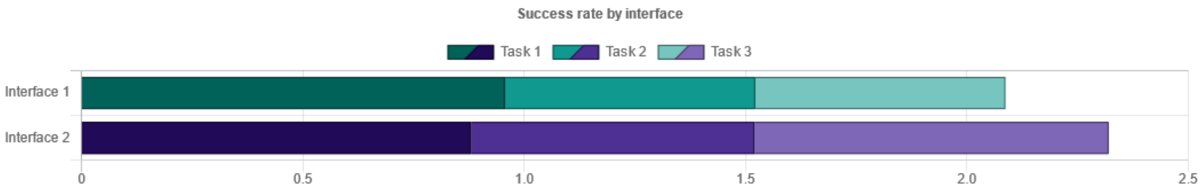

Figure S18. Task success rate chart stacked by interface for Generation 2.

Table S28. Success rate by interface for Generation 2.

	Interface 1						Interface 2						Mann-Whitney U test	
	Mean	Std. Dev.	N	Conf.	Conf. min.	Conf. max.	Mean	Std. Dev.	N	Conf.	Conf. min.	Conf. max.	P-value	H_0 rejection (α=0.05)
Task 1	0.9565	0.2085	23	0.0852	0.8713	1.0417	0.88	0.3317	25	0.13	0.75	1.01	0.3541	0
Task 2	0.5652	0.5069	23	0.2072	0.3581	0.7724	0.64	0.4899	25	0.192	0.448	0.832	0.609	0
Task 3	0.5652	0.5069	23	0.2072	0.3581	0.7724	0.8	0.4082	25	0.16	0.64	0.96	0.08508	0
All	0.6957	0.4635	69	0.1094	0.5863	0.805	0.7733	0.4215	75	0.0954	0.6779	0.8687	0.2936	0

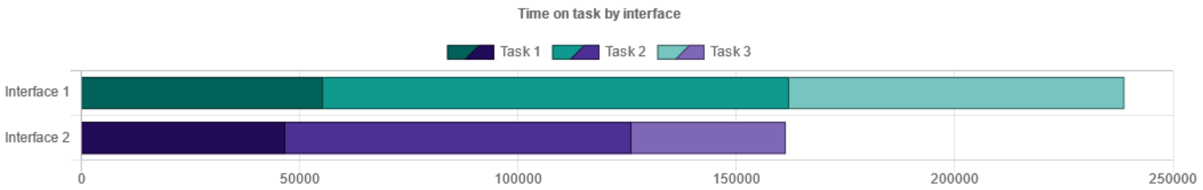

Figure S19. Time on task rate chart stacked by interface for Generation 2.

Table S29. Time on task by interface for Generation 2.

	Interface 1						Interface 2						Mann-Whitney U test	
	Mean	Std. Dev.	N	Conf.	Conf. min.	Conf. max.	Mean	Std. Dev.	N	Conf.	Conf. min.	Conf. max.	P-value	H_0 rejection (α=0.05)
Task 1	55,409.5652	82,295.1316	23	33,633.0534	21,776.5118	89,042.6186	46,644.92	49,370.569	25	19,353.263	27,291.657	65,998.183	0.4328	0
Task 2	106,691.4348	121,049.2802	23	49,471.4187	57,220.0161	156,162.8535	79,300.72	108,658.1587	25	42,593.9982	36,706.7218	121,894.7182	0.08662	0
Task 3	76,766.0435	69,141.1476	23	28,257.1747	48,508.8687	105,023.2182	35,364.16	31,053.8651	25	12,173.1151	23,191.0449	47,537.2751	0.02192	1
All	79,622.3478	94,484.254	69	22,294.1523	57,328.1955	101,916.5002	53,769.9333	72,692.9278	75	16,451.9583	37,317.975	70,221.8917	0.004634	1

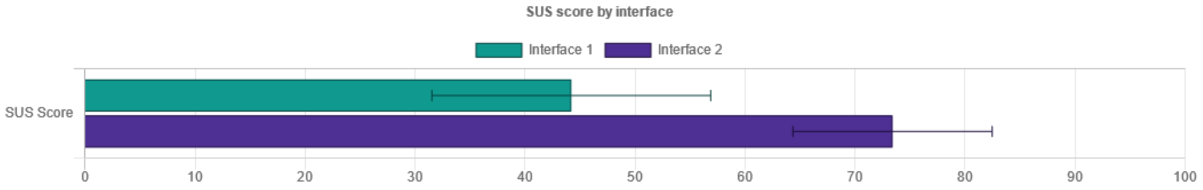

Figure S20. SUS score by interface chart for Generation 2.

Table S30. SUS score by interface for Generation 2.

	Interface 1						Interface 2						Mann-Whitney U test	
	Mean	Std. Dev.	N	Conf.	Conf. min.	Conf. max.	Mean	Std. Dev.	N	Conf.	Conf. min.	Conf. max.	P-value	H_0 rejection (α=0.05)
SUS Score	44.2045	30.3294	22	12.6739	31.5307	56.8784	73.4091	21.6787	22	9.059	64.3501	82.4681	0.002404	1

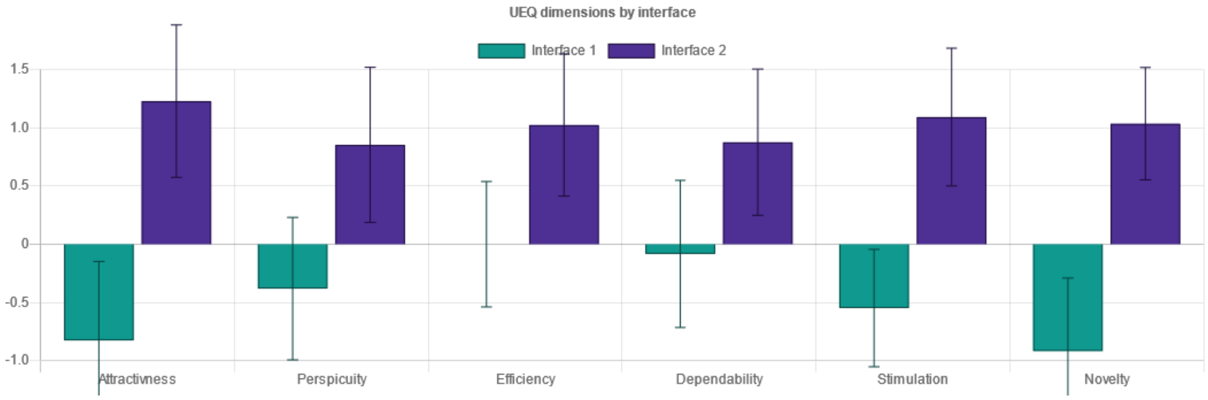

Figure S21. UEQ results by interface chart for Generation 2.

Table S31. UEQ results by interface for Generation 2.

Item	Interface 1						Interface 2						Mann-Whitney U test	
	Mean	Std. Dev.	N	Conf.	Conf. min.	Conf. max.	Mean	Std. Dev.	N	Conf.	Conf. min.	Conf. max.	P-value	H_0 rejection (α=0.05)
Attractivness	−0.8254	1.5842	21	0.6776	−1.503	−0.1478	1.2273	1.5648	22	0.6539	0.5734	1.8812	0.0003404	1
Perspicuity	−0.381	1.4288	21	0.6111	−0.992	0.2301	0.8523	1.5918	22	0.6652	0.1871	1.5174	0.01533	1
Efficiency	0	1.2575	21	0.5378	−0.5378	0.5378	1.0227	1.4596	22	0.6099	0.4128	1.6327	0.009041	1
Dependability	−0.0833	1.4755	21	0.6311	−0.7144	0.5477	0.875	1.5015	22	0.6274	0.2476	1.5024	0.06683	0
Stimulation	−0.5476	1.1769	21	0.5034	−1.051	−0.0442	1.0909	1.4133	22	0.5906	0.5003	1.6815	0.0006983	1
Novelty	−0.9167	1.4649	21	0.6265	−1.5432	−0.2901	1.0341	1.1529	22	0.4818	0.5523	1.5159	0.0001253	1

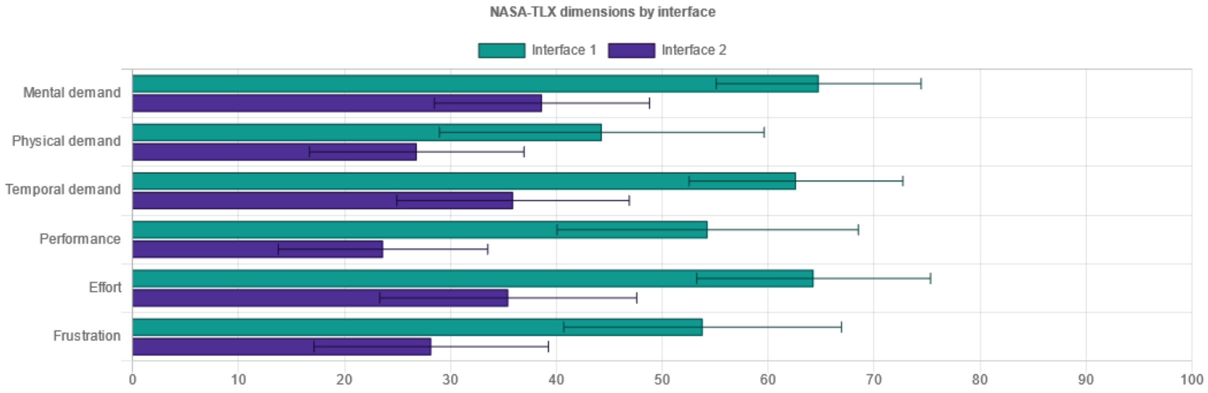

Figure S22. NASA-Task Load Index results by interface chart for Generation 2.

Table S32. NASA-Task Load results by interface for Generation 2.

Item	Interface 1						Interface 2						Mann-Whitney U test	
	Mean	Std. Dev.	N	Conf.	Conf. min.	Conf. max.	Mean	Std. Dev.	N	Conf.	Conf. min.	Conf. max.	P-value	H_0 rejection (α=0.05)
Mental demand	64.7619	22.6095	21	9.6703	55.0917	74.4322	38.6364	24.3086	22	10.1579	28.4784	48.7943	0.0009371	1
Physical demand	44.2857	35.822	21	15.3213	28.9644	59.607	26.8182	24.2284	22	10.1244	16.6938	36.9426	0.1068	0
Temporal demand	62.619	23.5913	21	10.0901	52.5289	72.7092	35.9091	26.2604	22	10.9735	24.9356	46.8826	0.001727	1
Performance	54.2857	33.2523	21	14.2222	40.0635	68.508	23.6364	23.6634	22	9.8883	13.7481	33.5247	0.003628	1
Effort	64.2857	25.8014	21	11.0355	53.2503	75.3212	35.4545	29.0283	22	12.1301	23.3244	47.5847	0.003689	1
Frustration	53.8095	30.6555	21	13.1116	40.6979	66.9211	28.1818	26.482	22	11.0661	17.1157	39.248	0.008767	1

8. Interface 1 (original) by Generations

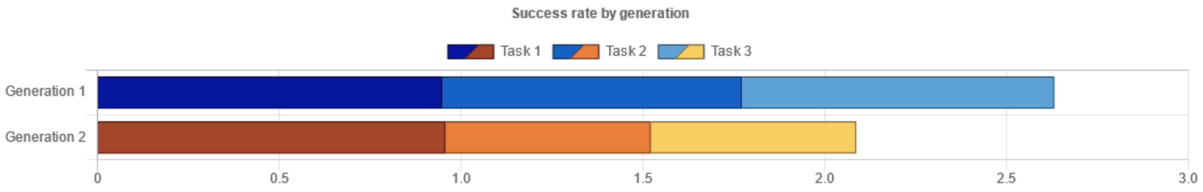

Figure S23. Task success rate chart stacked by generation for Interface 1.

Table S33. Success rate by by generation for Interface 1.

	Generation 1						Generation 2						Mann-Whitney U test	
	Mean	Std. Dev.	N	Conf.	Conf. min.	Conf. max.	Mean	Std. Dev.	N	Conf.	Conf. min.	Conf. max.	P-value	H_0 rejection (α=0.05)
Task 1	0.9474	0.2253	57	0.0585	0.8889	1.0059	0.9565	0.2085	23	0.0852	0.8713	1.0417	0.8769	0
Task 2	0.8246	0.3837	57	0.0996	0.7249	0.9242	0.5652	0.5069	23	0.2072	0.3581	0.7724	0.01629	1
Task 3	0.8596	0.3504	57	0.091	0.7687	0.9506	0.5652	0.5069	23	0.2072	0.3581	0.7724	0.004669	1
All	0.8772	0.3292	171	0.0493	0.8279	0.9265	0.6957	0.4635	69	0.1094	0.5863	0.805	0.0008334	1

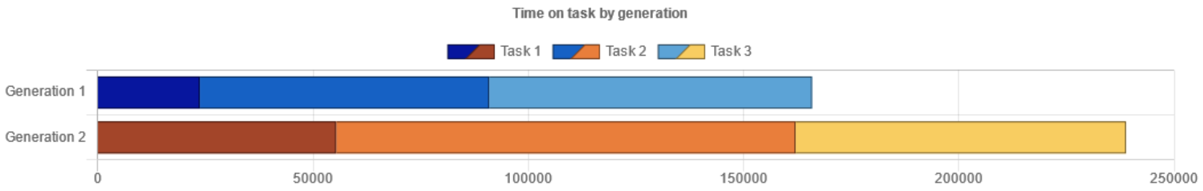

Figure S24. Time on task rate chart stacked by generation for Interface 1.

Table S34. Time on task by by generation for Interface 1.

	Generation 1						Generation 2						Mann-Whitney U test	
	Mean	Std. Dev.	N	Conf.	Conf. min.	Conf. max.	Mean	Std. Dev.	N	Conf.	Conf. min.	Conf. max.	P-value	H_0 rejection (α=0.05)
Task 1	23,717.6842	23,045.7753	57	5,982.8755	17,734.8087	29,700.5597	55,409.5652	82,295.1316	23	33,633.0534	21,776.5118	89,042.6186	0.0001268	1
Task 2	67,215.5088	85,927.6448	57	22,307.5334	44,907.9754	89,523.0422	10,6691.4348	121,049.2802	23	49,471.4187	57,220.0161	156,162.8535	0.1497	0
Task 3	75,014.5965	182,396.3249	57	47,351.6075	27,662.989	122,366.204	76,766.0435	69,141.1476	23	28,257.1747	48,508.8687	105,023.2182	0.3333	0
All	55,315.9298	118,653.0736	171	17,784.3095	375,31.6203	73,100.2394	79,622.3478	94,484.254	69	22,294.1523	57,328.1955	101,916.5002	0.001462	1

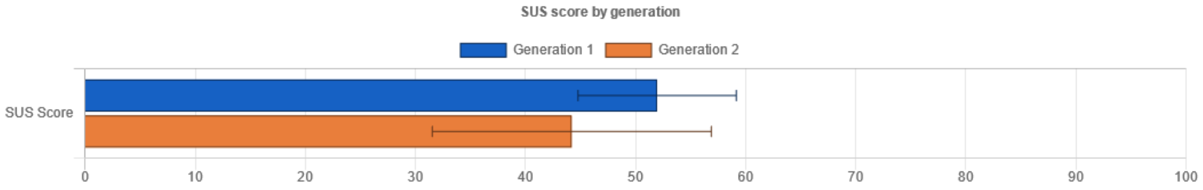

Figure S25. SUS score by generation for Interface 1.

Table S35. SUS score by generation for Interface 1.

	Generation 1						Generation 2						Mann-Whitney U test	
	Mean	Std. Dev.	N	Conf.	Conf. min.	Conf. max.	Mean	Std. Dev.	N	Conf.	Conf. min.	Conf. max.	P-value	H_0 rejection (α=0.05)
SUS Score	51.9608	26.1931	51	7.1888	44.772	59.1496	44.2045	30.3294	22	12.6739	31.5307	56.8784	0.3238	0

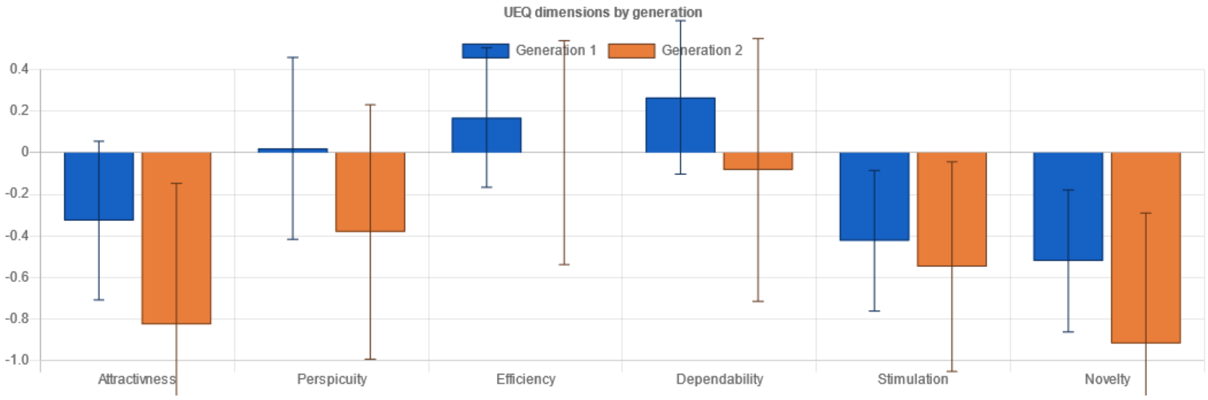

Figure S26. UEQ results by generation chart for Interface 1.

Table S36. UEQ results by generation for Interface 1.

Item	Generation 1						Generation 2						Mann-Whitney U test	
	Mean	Std. Dev.	N	Conf.	Conf. min.	Conf. max.	Mean	Std. Dev.	N	Conf.	Conf. min.	Conf. max.	P-value	H_0 rejection (α=0.05)
Attractivness	−0.3265	1.3612	49	0.3811	−0.7077	0.0546	−0.8254	1.5842	21	0.6776	−1.503	−0.1478	0.2107	0
Perspicuity	0.0204	1.5603	49	0.4369	−0.4165	0.4573	−0.381	1.4288	21	0.6111	−0.992	0.2301	0.3037	0
Efficiency	0.1684	1.1961	49	0.3349	−0.1665	0.5033	0	1.2575	21	0.5378	−0.5378	0.5378	0.5243	0
Dependability	0.2653	1.3164	49	0.3686	−0.1033	0.6339	−0.0833	1.4755	21	0.6311	−0.7144	0.5477	0.5234	0
Stimulation	−0.4235	1.2057	49	0.3376	−0.7611	−0.0859	−0.5476	1.1769	21	0.5034	−1.051	−0.0442	0.75	0
Novelty	−0.5204	1.2182	49	0.3411	−0.8615	−0.1793	−0.9167	1.4649	21	0.6265	−1.5432	−0.2901	0.2162	0

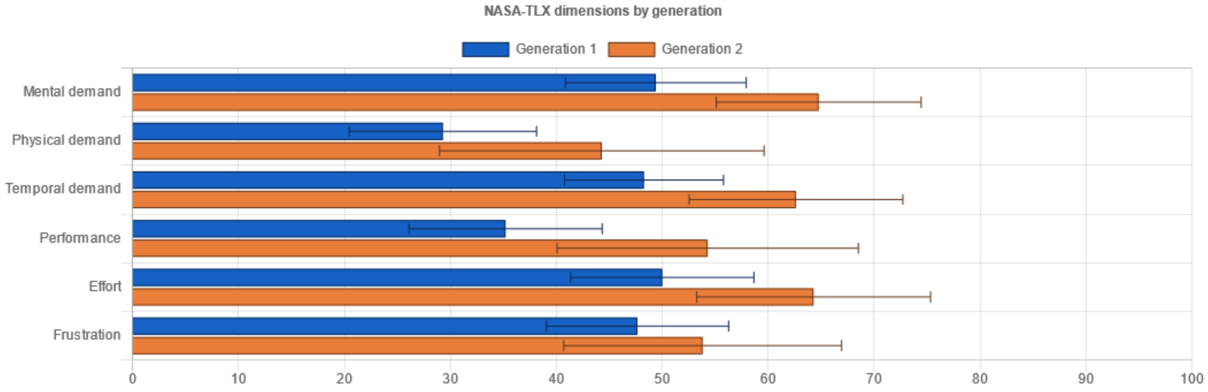

Figure S27. NASA-Task Load Index results by generation chart for Interface 1.

Table S37. NASA-Task Load results by generation for Interface 1.

Item	Generation 1						Generation 2						Mann-Whitney U test	
	Mean	Std. Dev.	N	Conf.	Conf. min.	Conf. max.	Mean	Std. Dev.	N	Conf.	Conf. min.	Conf. max.	P-value	H_0 rejection (α=0.05)
Mental demand	49.3878	30.493	49	8.5381	40.8497	57.9258	64.7619	22.6095	21	9.6703	55.0917	74.4322	0.04407	1
Physical demand	29.2857	31.5733	49	8.8405	20.4452	38.1262	44.2857	35.822	21	15.3213	28.9644	59.607	0.1166	0
Temporal demand	48.2653	26.8202	49	7.5097	40.7556	55.775	62.619	23.5913	21	10.0901	52.5289	72.7092	0.03308	1
Performance	35.2041	32.5634	49	9.1177	26.0863	44.3218	54.2857	33.2523	21	14.2222	40.0635	68.508	0.01987	1
Effort	50	30.8896	49	8.6491	41.3509	58.6491	64.2857	25.8014	21	11.0355	53.2503	75.3212	0.06309	0
Frustration	47.6531	30.7392	49	8.607	39.0461	56.26	53.8095	30.6555	21	13.1116	40.6979	66.9211	0.418	0

9. Interface 2 (FCD based) by Generations

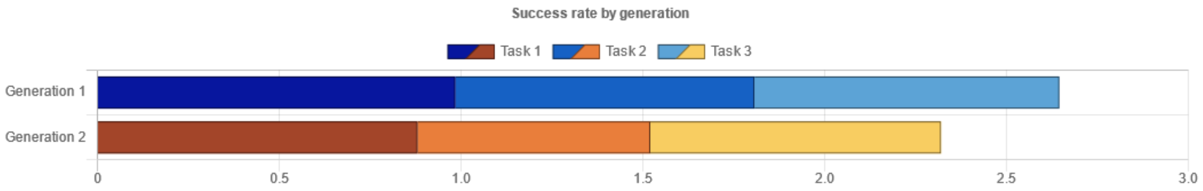

Figure S28. Task success rate chart stacked by generation for Interface 2.

Table S38. Success rate by generation for Interface 2.

	Generation 1						Generation 2						Mann-Whitney U test	
	Mean	Std. Dev.	N	Conf.	Conf. min.	Conf. max.	Mean	Std. Dev.	N	Conf.	Conf. min.	Conf. max.	P-value	H_0 rejection (α=0.05)
Task 1	0.9839	0.127	62	0.0316	0.9523	1.0155	0.88	0.3317	25	0.13	0.75	1.01	0.0386	1
Task 2	0.8226	0.3851	62	0.0959	0.7267	0.9185	0.64	0.4899	25	0.192	0.448	0.832	0.06959	0
Task 3	0.8387	0.3708	62	0.0923	0.7464	0.931	0.8	0.4082	25	0.16	0.64	0.96	0.6724	0
All	0.8817	0.3238	186	0.0465	0.8352	0.9283	0.7733	0.4215	75	0.0954	0.6779	0.8687	0.02663	1

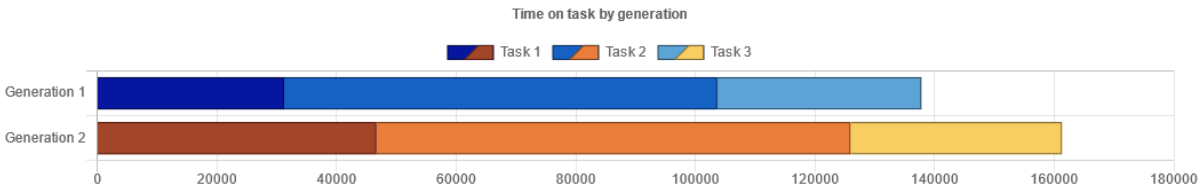

Figure S29. Time on task rate chart stacked by generation for Interface 2.

Table S39. Time on task by by generation for Interface 2.

	Generation 1						Generation 2						Mann-Whitney U test	
	Mean	Std. Dev.	N	Conf.	Conf. min.	Conf. max.	Mean	Std. Dev.	N	Conf.	Conf. min.	Conf. max.	P-value	H_0 rejection (α=0.05)
Task 1	31,224.6452	65,222.9093	62	16,235.3028	14,989.3423	47,459.948	46,644.92	49,370.569	25	19,353.263	27,291.657	65,998.183	0.06737	0
Task 2	72,428.1613	60,204.4881	62	14,986.1162	57,442.0451	87,414.2775	79,300.72	108,658.1587	25	42,593.9982	36,706.7218	121,894.7182	0.2524	0
Task 3	34,153.3065	26,791.5876	62	6,668.9687	27,484.3378	40,822.2751	35,364.16	31,053.8651	25	12,173.1151	23,191.0449	47,537.2751	0.9551	0
All	45,935.371	56,469.0708	186	8,115.4058	37,819.9652	54,050.7768	53,769.9333	72,692.9278	75	16,451.9583	37,317.975	70,221.8917	0.9285	0

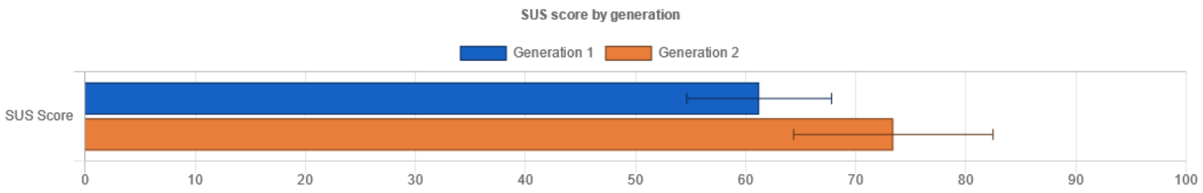

Figure S30. SUS score by generation for Interface 2.

Table S40. SUS score by generation for Interface 2.

	Generation 1						Generation 2						Mann-Whitney U test	
	Mean	Std. Dev.	N	Conf.	Conf. min.	Conf. max.	Mean	Std. Dev.	N	Conf.	Conf. min.	Conf. max.	P-value	H_0 rejection (α=0.05)
SUS Score	61.2264	24.4341	53	6.5783	54.6481	67.8047	73.4091	21.6787	22	9.059	64.3501	82.4681	0.04851	1

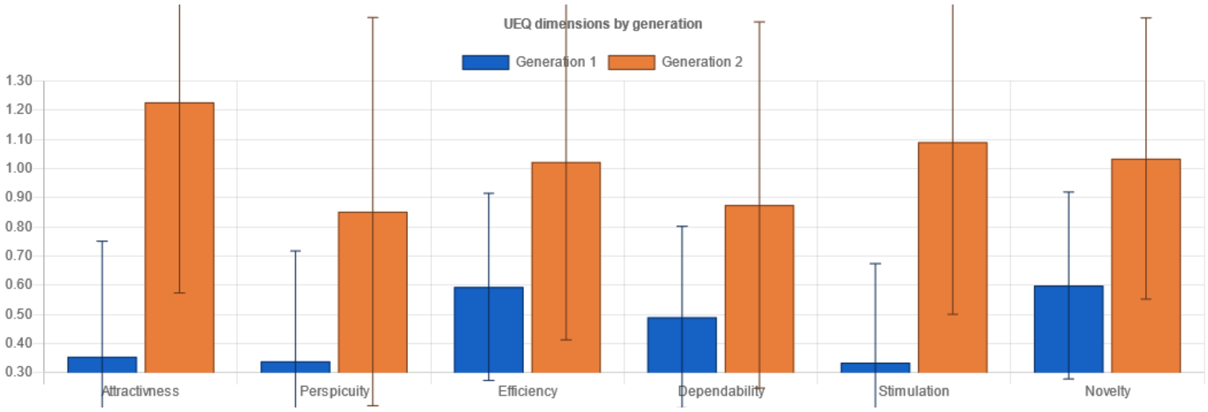

Figure S31. UEQ results by generation chart for Interface 2.

Table S41. UEQ results by generation for Interface 2.

Item	Generation 1						Generation 2						Mann-Whitney U test	
	Mean	Std. Dev.	N	Conf.	Conf. min.	Conf. max.	Mean	Std. Dev.	N	Conf.	Conf. min.	Conf. max.	P-value	H_0 rejection (α=0.05)
Attractivness	0.3553	1.4687	53	0.3954	−0.0401	0.7508	1.2273	1.5648	22	0.6539	0.5734	1.8812	0.01791	1
Perspicuity	0.3396	1.4032	53	0.3778	−0.0382	0.7174	0.8523	1.5918	22	0.6652	0.1871	1.5174	0.1544	0
Efficiency	0.5943	1.1911	53	0.3207	0.2737	0.915	1.0227	1.4596	22	0.6099	0.4128	1.6327	0.1583	0
Dependability	0.4906	1.156	53	0.3112	0.1793	0.8018	0.875	1.5015	22	0.6274	0.2476	1.5024	0.2291	0
Stimulation	0.3349	1.2595	53	0.3391	−0.0042	0.674	1.0909	1.4133	22	0.5906	0.5003	1.6815	0.03424	1
Novelty	0.5991	1.1892	53	0.3202	0.2789	0.9192	1.0341	1.1529	22	0.4818	0.5523	1.5159	0.2736	0

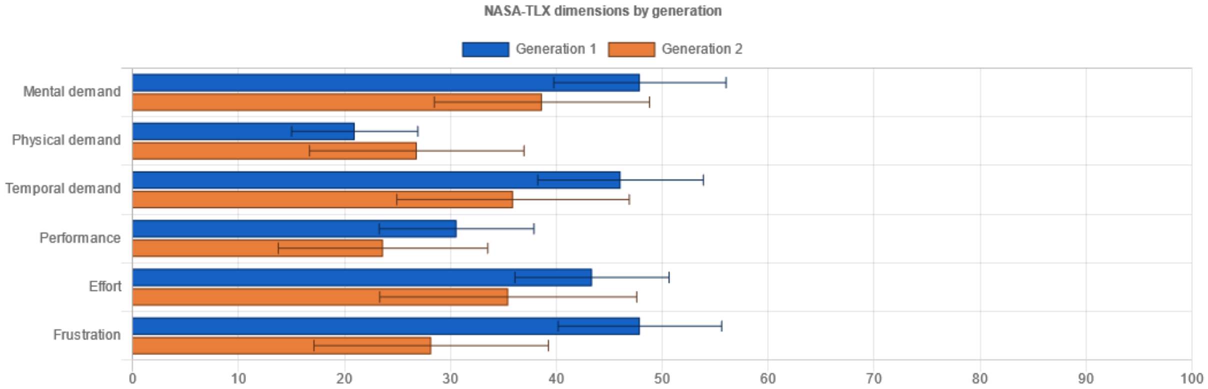

Figure S32. NASA-Task Load Index results by generation chart for Interface 2.

Table S42. NASA-Task Load results by generation for Interface 2.

Item	Generation 1						Generation 2						Mann-Whitney U test	
	Mean	Std. Dev.	N	Conf.	Conf. min.	Conf. max.	Mean	Std. Dev.	N	Conf.	Conf. min.	Conf. max.	P-value	H_0 rejection (α=0.05)
Mental demand	47.8846	29.9239	52	8.1334	39.7512	56.018	38.6364	24.3086	22	10.1579	28.4784	48.7943	0.2224	0
Physical demand	20.9615	21.9187	52	5.9576	15.004	26.9191	26.8182	24.2284	22	10.1244	16.6938	36.9426	0.3212	0
Temporal demand	46.0577	28.7541	52	7.8155	38.2422	53.8732	35.9091	26.2604	22	10.9735	24.9356	46.8826	0.2672	0
Performance	30.5769	26.874	52	7.3044	23.2725	37.8813	23.6364	23.6634	22	9.8883	13.7481	33.5247	0.3787	0
Effort	43.3654	26.7655	52	7.275	36.0904	50.6403	35.4545	29.0283	22	12.1301	23.3244	47.5847	0.2503	0
Frustration	47.8846	28.394	52	7.7176	40.167	55.6022	28.1818	26.482	22	11.0661	17.1157	39.248	0.007147	1
